# Enhance, delete, incept: Manipulating hippocampus-dependent memories^☆^

**DOI:** 10.1016/j.brainresbull.2013.12.011

**Published:** 2014-06

**Authors:** Hugo J. Spiers, Daniel Bendor

**Affiliations:** Institute of Behavioural Neuroscience, Research Department of Cognitive, Perceptual and Brain Sciences, Division of Psychology and Language Sciences, University College London, 26 Bedford Way, London WC1H 0AP, UK

**Keywords:** Memory consolidation, Sleep, Hippocampus, Fear conditioning, Extinction, Reconsolidation

## Abstract

Here we provide a brief overview of recent research on memory manipulation. We focus primarily on memories for which the hippocampus is thought to be required due to its central importance in the study of memory. The repertoire of methods employed is expanding and includes optogenetics, transcranial stimulation, deep brain stimulation, cued reactivation during sleep and the use of pharmacological agents. In addition, the possible mechanisms underlying these memory changes have been investigated using techniques such as single unit recording and functional magnetic resonance imaging (fMRI).

This article is part of a Special Issue entitled ‘Memory enhancement’.

## Highlights

• Newly developed methods allow manipulation of hippocampus-dependent memories.
• Cued reactivation during sleep and transcranial stimulation can enhance memories.
• Pharmacological agents can delete memories.
• Optogenetics and DREADDs can be used to incept memories.
• Electrophysiology and fMRI have been used to investigate the underlying mechanisms.

Our memories define us. They feed our grandest ambitions, lie at the root of our darkest fears, and allow us to travel in our mind from the present to the past. The idea that our precious memories might be susceptible to manipulation via external stimuli, such as leading questions in an interview, has long been known (Loftus and Palmer, 1974). However, a variety of technical advances have enabled researchers to manipulate memories more precisely and in new and innovative ways. Enhancing recall, deleting knowledge of the past and implanting fictitious memories – once the preserve of Hollywood blockbusters – are now becoming a reality.

Being able to manipulate memories has many potential benefits. Enhancing memory in patients afflicted with diseases such as Alzheimer's dementia opens a pathway to a substantially increased quality of life. Similarly, the capacity to dampen the impact of specific memories in conditions such as post-traumatic stress disorder (PTSD), phobias or anxiety disorders may provide a powerful means of potential treatment. Despite this, memory manipulation also has a dark-side. Films such as *Inception*, *The Eternal Sunshine of the Spotless Mind, Limitless, Total Recall* and *The Manchurian Candidate* provide prophetic warnings of the dangers of recklessly tampering with memories (see Appendix). While the ethics of memory manipulation are hotly debated (Liao and Sandberg, 2008; Mohamed and Sahakian, 2012; Ragan et al., 2013) research continues to advance at an ever-increasing pace.

Here we provide a brief overview of recent research on memory manipulation. We focus primarily on memories for which the hippocampus is thought to be required due to its central importance in the study of memory (Eichenbaum, 2004; Moscovitch et al., 2006; Squire et al., 2004; Spiers, 2012). The repertoire of methods employed is expanding and includes optogenetics (e.g. Ramirez et al., 2013), transcranial stimulation (e.g. Marshall et al., 2006), deep brain stimulation (e.g. Laxton et al., 2010), cued reactivation during sleep (e.g. Rasch et al., 2007; Rudoy et al., 2009) and the use of pharmacological agents (e.g. Steckler and Risbrough, 2012; De Kleine et al., 2013). In addition, the possible mechanisms underlying these memory changes have been investigated using techniques such as single unit recording (e.g. Bendor and Wilson, 2012) and functional magnetic resonance imaging (fMRI) (e.g. Hauner et al., 2013).

## 1 If only I could remember…

We all want to have a better memory. Why spend hours or even days studying for an exam, if with a photographic memory we could store this information almost instantaneously. While research has explored the enhancement of memory consolidation by the administration of putative ‘cognitive enhancers’ (e.g. Kaplan and Moore, 2011; Rodríguez et al., 2013) recent interest has focused on manipulating memories during sleep, tapping into the brain's normal memory consolidation process. Our brain processes a high volume of information every day, and to avoid being overwhelmed with the storage of all of this information, our brain must retain only a subset of our experiences. Each memory's storage must be prioritized according to its importance (e.g. your baby's first laugh should hopefully outrank the sound of the fan in the background). This process of selectively stabilizing specific memories is thought to occur most effectively during sleep (Stickgold and Walker, 2013). In particular, memory consolidation (Dudai, 2004; Frankland and Bontempi, 2005; Squire and Alvarez, 1995) for hippocampus-dependent learning (e.g. spatial association and word pairings) benefits specifically from the non-REM stage of sleep (Diekelmann and Born, 2010). While getting a good night of sleep will improve your memory, further memory enhancement is potentially possible if we can modify this sleep-consolidation process, and direct it towards specific memories.

To do this, we can take advantage of the fact that non-REM sleep has a number of signature rhythmic components that distinguish it from both an awake/active state and a REM sleep state (Buzsaki, 2009). The first rhythmic component is a slow wave oscillation – a large amplitude and low frequency (<1 Hz) variation in the local field potential (LFP) generated by the alternation between up and down states in neocortex (Buzsáki et al., 2012). The second rhythmic component is a spindle – a brief thalamocortical oscillation (7–14 Hz) – generated by the thalamic reticular nucleus (Steriade et al., 1993). Both of these non-REM sleep specific brain rhythms have been postulated to be important for memory consolidation, and therefore based on this idea, boosting the strength or increasing the amount of these oscillations could lead to memory enhancement. To boost slow wave oscillations, Marshall et al. (2006) applied a low frequency time-varying transcranial stimulation (0.75 Hz) to the frontal cortex of human subjects during early non-REM sleep. Interestingly, as a consequence of this low frequency transcranial stimulation, spindle power also increased. After being trained on a hippocampus-dependent task (word-pair associations), subjects went to sleep while receiving transcranial stimulation. When the subjects woke up, they were tested on the task, and the subjects that had received low frequency stimulation during sleep had better task performance (compared to control subjects that had received a sham stimulation). As this method boosted both slow-wave oscillations and spindles, both could potentially linked to this memory enhancement. More recently, optogenetics has been used to artificially induce spindles in rodents (Halassa et al., 2011), potentially providing a more effective (albeit invasive) method of boosting spindle production during sleep. Whether this methodology can be used during sleep to boost memory has not yet been demonstrated, nor is optogenetics currently viable for humans. Nonetheless, some traction has been achieved from invasive deep brain stimulation (DBS) in humans. DBS typically involves placing electrodes in deep neural structures for the treatment of severe dementias or obesity. In this method a continual stream of stimulation is applied to the nuclei or fibre tracks targeted. Electrodes stimulating the fornix and hypothalamus (Hamani et al., 2008; Laxton et al., 2010) or entorhinal cortex (Suthana et al., 2012) have been found to provide memory enhancement, perhaps paving the way for enhanced treatment. Whether stimulating during sleep has an added benefit over awake-stimulation has not yet been explored.

Another signature oscillation produced during non-REM sleep is the sharp-wave ripple, a 100–300 Hz brief oscillation generated within the hippocampus, and temporally correlated with spindle oscillations in prefrontal cortex (Siapas and Wilson, 1998). One interesting phenomenon related to sharp-wave ripples is replay, where sequential neural patterns associated to a previous behavioural episode spontaneously reactivate in the hippocampus and neocortex during a sharp-wave ripple event (Wilson and McNaughton, 1994; Lee and Wilson, 2002; Ji and Wilson, 2006). Replay events are a memory trace of a previous experience; replaying a memory trace again and again is a potential mechanism by which this memory could be reinforced and gradually consolidated. Blocking hippocampal sharp waves (which in turn silences replay events) leads to a memory impairment (Girardeau et al., 2009; Ego-Stengel and Wilson, 2010), which suggests that sharp wave ripple events and/or replay events may be important for memory consolidation. In order to improve specific memories using sharp wave ripples, can we manipulate the hippocampus to control what is getting replayed in the brain? One approach that has been used is pairing a sensory cue with a task, and then repeating this cue to the sleeping subject. When rats are trained on an auditory-spatial association task (each sound is associated with a particular reward location), playing these cues bias replay events towards replaying the experience associated with the cue (Bendor and Wilson, 2012). So if we can bias replay towards a particular memory when we sleep, can we use this to boost memories? In humans, if the same sensory cue (olfactory or auditory) is present during the training and afterwards during non-REM sleep, task performance is enhanced during the post-nap test (i.e. less forgetting) (Rasch et al., 2007; Rudoy et al., 2009; Diekelmann et al., 2011; Antony et al., 2012; Rolls et al., 2013). This method of targeted memory reactivation (Oudiette and Paller, 2013) only works during non-REM sleep; no memory improvement occurs for cue presentation during the awake state or during REM sleep (Rasch et al., 2007; Diekelmann et al., 2011).

Thus far, we have discussed three methods (transcranial stimulation, deep brain stimulation and targeted memory reactivation) that have the potential to enhance the strength of memories or bias the memory trace content during non-REM sleep specific brain rhythms. While these methods lead to a memory boost, it is important to note that the effect, while statistically significant, is typically mild (∼10% improvement). The ability to manipulate multiple brain rhythms together (ripple–spindle interactions) and more precisely target the neural circuits of a particular memory (Liu et al., 2012) may produce a larger memory boost.

## 2 If only I could forget…

While a method for enhancing memories would seem an asset, a procedure for deleting memories comes laden with more nefarious connotations. The disastrous consequences of erasing memories from a broken relationship are played out in the film *The Eternal Sunshine of the Spotless Mind* (see Appendix). Amnesia for personally known individuals can occur in cases of semantic dementia (Thompson et al., 2004). However, given that semantic memories appear to be widely distributed in the neocortex (Martin and Chao, 2001; McClelland and Rogers, 2003) it is highly questionable that it would be possible to erase all the memories associated with a single individual. By contrast, disrupting memory for a single event, or learned association is not so far flung. Rather than something to be feared, memory disruption may prove substantially beneficial for the treatment of disorders such as PTSD, phobias and anxiety disorders.

While the brain may have developed specific mechanisms for modulating which memories are to be degraded (Frankland et al., 2013; Hardt et al., 2013), researchers have sought to improve this via the application of selected drugs. Pharmacological treatment of the persistent involuntary memory retrieval that accompanies PTSD has been explored in numerous studies (see e.g. Steckler and Risbrough, 2012; De Kleine et al., 2013 for reviews). In both clinical and laboratory settings, a wide variety of pharmacological agents have been used to modifying memories, with particular emphasis on disrupting fear-related memories (Kaplan and Moore, 2011). These have included targeting glucocorticoid (e.g. De Bitencourt et al., 2013), glutamtergic (Kuriyama et al., 2013), GABAergic (Rodríguez et al., 2013) adrenergic (Kindt et al., 2009), cannabinoid (Rabinak et al., 2013), serotonergic (Zhang et al., 2013) and glycine (File et al., 1999) receptors.

Research on disrupting memory derives predominately from studying Pavlovian fear conditioning using an electrical shock. In “auditory fear conditioning” a rodent is initially exposed to repeated pairings of an electrical shock with a neutral tone. This leads to the tone alone evoking a fearful memory of getting shocked, which results in freezing behaviour in the rodent (Maren, 2001). In a variant of this method, contextual fear conditioning, the animal is exposed to a novel environment, during which it receives one or more electric shocks resulting in a learned hippocampus-dependent association between the environmental context (instead of an auditory cue) and the potential for more shocks (Kim and Fanselow, 1992). With repeated exposure to the tone or the context alone the animal will eventually stop freezing. This is referred to as extinction. Infusion of fibroblast growth factor 2 (an agent affecting neural cell development and neurogenesis) into the amygdala immediately after extinction strongly increases the chance that this memory will not re-surface at a later time point (Graham and Richardson, 2011). Whether a similar approach targeting the hippocampus can be used to enhance contextual fear conditioning remains to be determined. Recent work has revealed that extinction of conditioned fear memories can be enhanced via re-activation of the memories during non-REM sleep. Hauner et al. (2013) conditioned humans to expect a shock when viewing certain faces. The presentation of the shocked faces was paired with certain odours. Later during non-REM sleep subjects were re-exposed to the odours associated with some of the feared faces. Conditioned responses to the faces associated with the odours that were re-presented during sleep were significantly less than those faces paired with odours not presented. The impact of the odour presentation was apparent in the reduction of hippocampal activity and re-organization of activity patterns in the amygdala when pre- and post-sleep conditioning periods were examined with fMRI, further highlighting the importance of these brain regions. Although these results appear to be contradictory to the memory-enhancing results obtained using cued-reactivation during non-REM sleep (Rasch et al., 2007; Rudoy et al., 2009; Rolls et al., 2013), the extinction of a fear memory is not necessarily caused by memory deletion. Rather, extinction likely involves the active suppression of a still intact fear memory by regions of the brain distinct from where the original fear memory is stored (Milad and Quirk, 2002).

While enhancing extinction is one means of suppressing a memory, another method is to manipulate the brain at a time point many weeks later when a stored memory has been re-activated and requires stabilizing, a process known as reconsolidation (Misanin et al., 1968; Sara, 2010; Dudai, 2004). Infusion of protein synthesis inhibitors during periods after re-activation of memory has been shown to strongly disrupt future memory expression (Nader et al., 2000). Studies investigated manipulating reconsolidation in humans have focused on the effects of the adrenergic modulator propranolol (Brunet et al., 2011a; Kindt et al., 2009). Because propranolol must be administered before the re-activation to have an effect, it has been debated as to whether reconsolidation processes have been specifically targeted (Brunet et al., 2011b) or not (Schiller and Phelps, 2011).

The maintenance of long-term potentiation (LTP), an activity-dependent, persistent form of synaptic plasticity, is a key model for memory storage at a cellular level (Malenka and Bear, 2004). LTP is a complex and heterogeneous phenomenon (beyond the scope of this review), however in a simplified model of LTP, synapses that have been active during an experience become strengthened to form a memory of that experience; The persistence of this memory then depends on the continued maintenance of LTP in these synapses. Previous work has suggested that persistent phosphorylation by PKMζ (protein kinase M zeta) is required for this maintenance (Ling et al., 2002), and the injection of ZIP (a synthetic ζ-pseudosubstrate inhibitory peptide) can inhibit PKMζ and disrupt LTP (Serrano et al., 2005). Injecting ZIP in the hippocampus of rats, one day after they are trained in an active place avoidance task, can permanently delete the spatial memory related to this task (Pastalkova et al., 2006). The ability of ZIP to delete a memory also extends to other brain regions outside of the hippocampus, including the deletion of a taste-aversion memory stored in the insula (Shema et al., 2007). As an alternative to using ZIP, a lentivirus-induced overexpression of a dominant-negative PKMζ mutation in insular cortex can also be used to block a taste-aversion memory (Shema et al., 2011). Interestingly, if a normal version of PKMζ is overexpressed in insular cortex (using the same lentiviral approach), this leads to a general enhancement in taste aversion memories (Shema et al., 2011). However, more recent evidence suggests that the relationship between ZIP, PKMζ, and maintenance of LTP may be more complicated. Transgenic mice lacking PKMζ have normal memory function, suggesting that PKMζ, however, may not be the only kinase involved in LTP maintenance (Volk et al., 2013; Lee et al., 2013). As ZIP is still effective in erasing memories in PKMζ null mice, ZIP does not require PKMζ to function, and the mechanism by which ZIP can erase memories remains an open question (Volk et al., 2013; Lee et al., 2013).

## 3 “I know kung fu”

Anything is possible in science fiction, including having the knowledge of kung fu “downloaded” into our brains, as the character Neo has done in the movie *The Matrix* (see Appendix). In reality, is it possible to artificially store or “incept” new memories in our brain? In addition to enhancing the memories of recent experiences using sleep-specific manipulations, it is also possible to form new memories in the brain during sleep, creating associations between two sensory stimuli that have not been previously experienced together by the awake subject. To do this, Arzi et al. (2012) presented tones paired with odours to sleeping subjects (e.g. a high frequency tone with an unpleasant odour). Pleasant odours evoke a larger sniff volume than unpleasant odours, and if there is an expectation of a particular odour, this can be observed in the size of the sniff volume accompanying the tone (when presented without the odour). If the odour-tone pairings were presented during non-REM sleep, subjects (after waking up) had larger sniff volumes for the tone associated with the pleasant odour, compared to the tone associated with the unpleasant odour. While these data demonstrate that we are capable of unconsciously learning new things (while asleep), the new associations formed are still the direct result of natural stimulation. To take these experiments one-step closer towards “downloading” information artificially, several groups have incorporated a strategy of using molecular genetic techniques to artificially target brain circuits associated with a particular memory. Neural circuits storing a memory can be targeted using a transgenic mouse (c-Fos-tTA), that has the tetracycline transactivator (tTA) under the control of the immediate early gene c-Fos. Because c-Fos expression is driven by recent neural activity, tTA can be limited spatially to the neural circuit activated by a recent experience, and temporally by the removal of doxycycline from the mouse's diet (presence of doxycycline inhibits the binding of tTA to its target). In combination with c-Fos-tTA, transgenic mice also had tTA-driven transcription of either ChR2 (channelrhodopsin) or an hM_3_D_q_ receptor (excitatory DREADDs) (Liu et al., 2012; Garner et al., 2012). Next, mice were introduced to a novel context, where they received several mild shocks, creating a contextual fear memory while the tTA was active. Now using either light (for ChR2 mice) or an IP injection of Clozapine-N-Oxide (hM_3_D_q_ mice), the neural circuit storing this fear memory could be activated, causing the mouse to freeze in a context that it had *not* been previously shocked. Because the DREADDs approach was non-specific to the brain regions targeted, it is likely that only a subset of the hM_3_D_q_ expressing neurons actually store the memory engram. In contrast, the optogenetics approach (Liu et al., 2012) only targeted the dentate gyrus, however whether the actual memory engram is stored in the ChR2 expressing neurons, or if it resides further downstream (e.g. CA3 and CA1 of the hippocampus) remains to be demonstrated.

To take this one step further and create an artificial memory, Ramirez and colleagues used the same approach (c-Fos-tTA mice expressing ChR2) to target the neural circuit encoding a context (Ramirez et al., 2013). Even though the mouse was never shocked in this context, after light stimulation (activating the memory of this context) was paired with a shock, the mouse would now freeze upon entry into the context. These data suggest that it is possible to artificially create a contextual fear memory. It is important to note that similar to sleep-dependent methods, this technique can only create new associations between already experienced events. While we are still far away from the ability to download kung fu into our brains, we have crossed this first hurdle of incepting new associative memories in our brains.

## 4 Conclusion

In this review, we have outlined a number of different methodologies for modifying hippocampus-dependent memories, that lead the way for new developments in memory enhancement, deletion, and even inception. One strategy is take advantage of brain state, as memories are more easily modified during sleep (Diekelmann and Born, 2010; Oudiette and Paller, 2013). Acting at a neuronal level, a second strategy relies on targeting specific neurons using molecular-genetic techniques, allowing external control over the neural circuits involved in encoding a specific memory (Liu et al., 2012; Garner et al., 2012). Acting at the synaptic level, a third strategy is to affect the cellular pathways involved in maintaining a memory (Pastalkova et al., 2006). As each strategy acts at a different level of the brain, combining these strategies together may ultimately lead to an even more effective method of modifying memories. While further research will lead to newer and better ways of enhancing, deleting, and incepting memories, whether science is able to one day “catch up” to science fiction remains to be seen.

## Appendix Movies about memory enhancement, deletion, and inception

**Eternal Sunshine of the Spotless Mind (2004):** After breaking up with his girlfriend, the main character has a procedure performed – while he sleeps, a machine zaps and deletes all the memories of his ex-girlfriend. This technology replaces more established gustatory-driven methods of recovering from a break-up, like eating several cartons of ice cream.

**Inception (2010):** Using a “shared dream” technology, the main character and his team attempt to implant false memories (inception) in an unsuspecting target. The larger question is how they got all that “dream-hacking” equipment through airport security?

**Limitless (2011):** The main character takes a mystery pill (NZT) that substantially enhancing his cognitive abilities. The movie demonstrates some of the downsides of “genius withdrawal”.

**The Manchurian Candidate (1962, 2004 [remake]):** A solider is brainwashed (conditioned without conscious memory) by the enemy to become an assassin. After receiving the trigger symbol (or spoken phrase), the solider unconsciously carriers out the instruction given to him (such as assassinating a target), forgetting everything related to these actions afterwards. Fortunately this is science fiction, and we haven’t all been brainwashed to execute a particular “triggered” action. Well with the exception of advertising…

**The Matrix Trilogy (1999, 2003):** The year is 2199. After a war between humans and computers, humans now live inside a virtual reality environment called “the Matrix”, where humans still think its 1999, and are unaware of what has happened. The few humans that have managed to leave the Matrix are staging a revolution, and must re-enter the Matrix to fight the computers. As the Matrix is essentially software, computer code structured by rules, humans find that it is possible to “download” new skills and learn to bend or even break the rules of physics. The writers also decide to break the rules of physics by ignoring the first law of thermodynamics, suggesting that humans within the Matrix are used as energy sources (producing more energy than they require to survive).

**Total Recall (1990):** Implanting a false memory of a vacation to Mars has bizarre consequences for the main character, unlocking a supressed memory of his true identity – a secret agent. Could this movie have be the inspiration behind Newt Gingrich's presidential campaign promise – to build a space colony on Mars?

**Total Recall (2012 [remake]):** A poorly done remake of the 1990 Total Recall movie. After watching this, you may want to look into some memory deletion technology (see Eternal Sunshine of the Spotless Mind).

See Baxendale (2004) for a review of movies exploring memory-related themes.

## References

[bib0005] Antony J.W., Gobel E.W., O’Hare J.K., Reber P.J., Paller K.A. (2012). Cued memory reactivation during sleep influences skill learning. Nature Neuroscience.

[bib0010] Arzi A., Shedlesky L., Ben-Shaul M., Nasser K., Oksenberg A., Hairston I.S., Sobel N. (2012). Humans can learn new information during sleep. Nature Neuroscience.

[bib0015] Bendor D., Wilson M.A. (2012). Biasing the content of hippocampal replay during sleep. Nature Neuroscience.

[bib0020] Baxendale S. (2004). Memories aren’t made of this: amnesia at the movies. BMJ: British Medical Journal.

[bib0345] Brunet A., Poundja J., Tremblay J., Bui E., Thomas E., Orr S.P., Azzoug A., Birmes P., Pitman R.K. (2011). Trauma reactivation under the influence of propranolol decreases posttraumatic stress symptoms and disorder: 3 open-label trials. Journal of Clinical Psychopharmacology.

[bib0350] Brunet A., Ashbaugh A.R., Saumier D., Pitman R.K., Nelson M., Tremblay J., Roullet P., Birmes P. (2011). Does reconsolidation occur in humans: a reply. Frontiers in Behavioral Neuroscience.

[bib0025] Buzsaki G. (2009). Rhythms of the Brain.

[bib0030] Buzsáki G., Anastassiou C.A., Koch C. (2012). The origin of extracellular fields and currents—EEG, ECoG, LFP and spikes. Nature Reviews Neuroscience.

[bib0035] De Bitencourt R.M., Pamplona F.A., Takahashi R.N. (2013). A current overview of cannabinoids and glucocorticoids in facilitating extinction of aversive memories: potential extinction enhancers. Neuropharmacology.

[bib0040] De Kleine R.A., Rothbaum B.O., van Minnen A. (2013). Pharmacological enhancement of exposure-based treatment in PTSD: a qualitative review. European Journal of Pharmacology.

[bib0045] Diekelmann S., Born J. (2010). The memory function of sleep. Nature Reviews Neuroscience.

[bib0050] Diekelmann S., Büchel C., Born J., Rasch B. (2011). Labile or stable: opposing consequences for memory when reactivated during waking and sleep. Nature Neuroscience.

[bib0055] Dudai Y. (2004). The neurobiology of consolidations, or, how stable is the engram?. Annual Review of Psychology.

[bib0060] Eichenbaum H. (2004). Hippocampus: cognitive processes and neural representations that underlie declarative memory. Neuron.

[bib0065] Ego-Stengel V., Wilson M.A. (2010). Disruption of ripple associated hippocampal activity during rest impairs spatial learning in the rat. Hippocampus.

[bib0070] File S.E., Fluck E., Fernandes C. (1999). Beneficial effects of glycine (bioglycin) on memory and attention in young and middle-aged adults. Journal of Clinical Psychopharmacology.

[bib0075] Frankland P.W., Bontempi B. (2005). The organization of recent and remote memories. Nature Reviews Neuroscience.

[bib0355] Frankland P.W., Köhler S., Josselyn S.A. (2013). Hippocampal neurogenesis and forgetting. Trends in Neurosciences.

[bib0080] Garner A.R., Rowland D.C., Hwang S.Y., Baumgaertel K., Roth B.L., Kentros C., Mayford M. (2012). Generation of a synthetic memory trace. Science.

[bib0085] Girardeau G., Benchenane K., Wiener S.I., Buzsáki G., Zugaro M.B. (2009). Selective suppression of hippocampal ripples impairs spatial memory. Nature Neuroscience.

[bib0090] Graham B.M., Richardson R. (2011). Intraamygdala infusion of fibroblast growth factor 2 enhances extinction and reduces renewal and reinstatement in adult rats. Journal of Neuroscience.

[bib0095] Halassa M.M., Siegle J.H., Ritt J.T., Ting J.T., Feng G., Moore C.I. (2011). Selective optical drive of thalamic reticular nucleus generates thalamic bursts and cortical spindles. Nature Neuroscience.

[bib0100] Hamani C., McAndrews M.P., Cohn M., Oh M., Zumsteg D., Shapiro C.M., Lozano A.M. (2008). Memory enhancement induced by hypothalamic/fornix deep brain stimulation. Annals of Neurology.

[bib0360] Hardt O., Nader K., Nadel L. (2013). Decay happens: the role of active forgetting in memory. Trends in Cognitive Sciences.

[bib0105] Hauner K.K., Howard J.D., Zelano C., Gottfried J.A. (2013). Stimulus-specific enhancement of fear extinction during slow-wave sleep. Nature Neuroscience.

[bib0110] Ji D., Wilson M.A. (2006). Coordinated memory replay in the visual cortex and hippocampus during sleep. Nature Neuroscience.

[bib0115] Kaplan G.B., Moore K.A. (2011). The use of cognitive enhancers in animal models of fear extinction. Pharmacology Biochemistry and Behavior.

[bib0120] Kim J.J., Fanselow M.S. (1992). Modality-specific retrograde amnesia of fear. Science.

[bib0125] Kindt M., Soeter M., Vervliet B. (2009). Beyond extinction: erasing human fear responses and preventing the return of fear. Nature Neuroscience.

[bib0130] Kuriyama K., Honma M., Yoshiike T., Kim Y. (2013). Valproic acid but not d-cycloserine facilitates sleep-dependent offline learning of extinction and habituation of conditioned fear in humans. Neuropharmacology.

[bib0135] Laxton A.W., Tang-Wai D.F., McAndrews M.P., Zumsteg D., Wennberg R., Keren R., Lozano A.M. (2010). A phase I trial of deep brain stimulation of memory circuits in Alzheimer's disease. Annals of Neurology.

[bib0140] Lee A.K., Wilson M.A. (2002). Memory of sequential experience in the hippocampus during slow wave sleep. Neuron.

[bib0145] Lee A.M., Kanter B.R., Wang D., Lim J.P., Zou M.E., Qiu C., Messing R.O. (2013). Prkcz null mice show normal learning and memory. Nature.

[bib0150] Liao S.M., Sandberg A. (2008). The normativity of memory modification. Neuroethics.

[bib0155] Ling D.S., Benardo L.S., Serrano P.A., Blace N., Kelly M.T., Crary J.F., Sacktor T.C. (2002). Protein kinase Mζ is necessary and sufficient for LTP maintenance. Nature Neuroscience.

[bib0160] Liu X., Ramirez S., Pang P.T., Puryear C.B., Govindarajan A., Deisseroth K., Tonegawa S. (2012). Optogenetic stimulation of a hippocampal engram activates fear memory recall. Nature.

[bib0165] Loftus E.F., Palmer J.C. (1974). Reconstruction of automobile destruction: an example of the interaction between language and memory. Journal of Verbal Learning and Verbal Behavior.

[bib0170] Malenka R.C., Bear M.F. (2004). LTP and LTD: an embarrassment of riches. Neuron.

[bib0175] Maren S. (2001). Neurobiology of Pavlovian fear conditioning. Annual Review of Neuroscience.

[bib0180] Martin A., Chao L.L. (2001). Semantic memory and the brain: structure and processes. Current Opinion in Neurobiology.

[bib0185] Marshall L., Helgadóttir H., Mölle M., Born J. (2006). Boosting slow oscillations during sleep potentiates memory. Nature.

[bib0190] McClelland J.L., Rogers T.T. (2003). The parallel distributed processing approach to semantic cognition. Nature Reviews Neuroscience.

[bib0195] Milad M.R., Quirk G.J. (2002). Neurons in medial prefrontal cortex signal memory for fear extinction. Nature.

[bib0200] Misanin J.R., Miller R.R., Lewis D.J. (1968). Retrograde amnesia produced by electroconvulsive shock after reactivation of a consolidated memory trace. Science.

[bib0205] Mohamed A.D., Sahakian B.J. (2012). The ethics of elective psychopharmacology. International Journal of Neuropsychopharmacology: Official Scientific Journal of the Collegium Internationale Neuropsychopharmacologicum (CINP).

[bib0210] Moscovitch M., Nadel L., Winocur G., Gilboa A., Rosenbaum R.S. (2006). The cognitive neuroscience of remote episodic, semantic and spatial memory. Current Opinion in Neurobiology.

[bib0215] Nader K., Schafe G.E., Le Doux J.E. (2000). Fear memories require protein synthesis in the amygdala for reconsolidation after retrieval. Nature.

[bib0220] Oudiette D., Paller K.A. (2013). Upgrading the sleeping brain with targeted memory reactivation. Trends in Cognitive Sciences.

[bib0225] Pastalkova E., Serrano P., Pinkhasova D., Wallace E., Fenton A.A., Sacktor T.C. (2006). Storage of spatial information by the maintenance mechanism of LTP. Science.

[bib0230] Rabinak C.A., Angstadt M., Sripada C.S., Abelson J.L., Liberzon I., Milad M.R., Phan K.L. (2013). Cannabinoid facilitation of fear extinction memory recall in humans. Neuropharmacology.

[bib0235] Ragan C.I., Bard I., Singh I. (2013). What should we do about student use of cognitive enhancers? An analysis of current evidence. Neuropharmacology.

[bib0240] Ramirez S., Liu X., Lin P.A., Suh J., Pignatelli M., Redondo R.L., Tonegawa S. (2013). Creating a false memory in the hippocampus. Science.

[bib0245] Rasch B., Büchel C., Gais S., Born J. (2007). Odor cues during slow-wave sleep prompt declarative memory consolidation. Science.

[bib0250] Rodríguez M.L.C., Campos J., Forcato C., Leiguarda R., Maldonado H., Molina V.A., Pedreira M.E. (2013). Enhancing a declarative memory in humans: the effect of clonazepam on reconsolidation. Neuropharmacology.

[bib0255] Rolls A., Makam M., Kroeger D., Colas D., de Lecea L., Heller H.C. (2013). Sleep to forget: interference of fear memories during sleep. Molecular Psychiatry.

[bib0260] Rudoy J.D., Voss J.L., Westerberg C.E., Paller K.A. (2009). Strengthening individual memories by reactivating them during sleep. Science.

[bib0265] Sara S.J. (2010). Reactivation, retrieval, replay and reconsolidation in and out of sleep: connecting the dots. Frontiers in Behavioral Neuroscience.

[bib0365] Schiller D., Phelps E.A. (2011). Does reconsolidation occur in humans?. Frontiers in Behavioral Neuroscience.

[bib0270] Serrano P., Yao Y., Sacktor T.C. (2005). Persistent phosphorylation by protein kinase Mζ maintains late-phase long-term potentiation. Journal of Neuroscience.

[bib0275] Shema R., Sacktor T.C., Dudai Y. (2007). Rapid erasure of long-term memory associations in the cortex by an inhibitor of PKMζ. Science.

[bib0280] Shema R., Haramati S., Ron S., Hazvi S., Chen A., Sacktor T.C., Dudai Y. (2011). Enhancement of consolidated long-term memory by overexpression of protein kinase Mζ in the neocortex. Science.

[bib0285] Siapas A.G., Wilson M.A. (1998). Coordinated interactions between hippocampal ripples and cortical spindles during slow-wave sleep. Neuron.

[bib0290] Spiers H.J., Ramachandran V.S. (2012). Hippocampal formation.

[bib0295] Squire L.R., Alvarez P. (1995). Retrograde amnesia and memory consolidation: a neurobiological perspective. Current Opinion in Neurobiology.

[bib0300] Squire L.R., Stark C.E.L., Clark R.E. (2004). The medial temporal lobe. Annual Review of Neuroscience.

[bib0305] Steckler T., Risbrough V. (2012). Pharmacological treatment of PTSD-established and new approaches. Neuropharmacology.

[bib0310] Steriade M., McCormick D.A., Sejnowski T.J. (1993). Thalamocortical oscillations in the sleeping and aroused brain. Science.

[bib0315] Stickgold R., Walker M.P. (2013). Sleep-dependent memory triage: evolving generalization through selective processing. Nature Neuroscience.

[bib0320] Suthana N., Haneef Z., Stern J., Mukamel R., Behnke E., Knowlton B., Fried I. (2012). Memory enhancement and deep-brain stimulation of the entorhinal area. New England Journal of Medicine.

[bib0325] Thompson S.A., Graham K.S., Williams G., Patterson K., Kapur N., Hodges J.R. (2004). Dissociating person-specific from general semantic knowledge: roles of the left and right temporal lobes. Neuropsychologia.

[bib0330] Volk L.J., Bachman J.L., Johnson R., Yu Y., Huganir R.L. (2013). PKM-[zgr] is not required for hippocampal synaptic plasticity, learning and memory. Nature.

[bib0335] Wilson M.A., McNaughton B.L. (1994). Reactivation of hippocampal ensemble memories during sleep. Science.

[bib0340] Zhang G., Ásgeirsdóttir H.N., Cohen S.J., Munchow A.H., Barrera M.P., Stackman R.W. (2013). Stimulation of serotonin 2A receptors facilitates consolidation and extinction of fear memory in C57BL/6J mice. Neuropharmacology.

